# Synbiotic-like effect of linoleic acid overproducing *Lactobacillus casei* with berry phenolic extracts against pathogenesis of enterohemorrhagic *Escherichia coli*

**DOI:** 10.1186/s13099-019-0320-y

**Published:** 2019-07-27

**Authors:** Zajeba Tabashsum, Mengfei Peng, Cassendra Bernhardt, Puja Patel, Michael Carrion, Debabrata Biswas

**Affiliations:** 1 Biological Sciences Program-Molecular and Cellular Biology, University of Maryland, College Park, MD USA; 2 Department of Animal and Avian Sciences, University of Maryland, 8127 Regents Drive, College Park, MD USA; 3 Center for Food Safety and Security Systems, University of Maryland, College Park, MD USA

**Keywords:** EHEC, Prebiotic, Probiotic, Synbiotic, Prevention

## Abstract

**Background:**

Majority of enteric infections are foodborne and antimicrobials including antibiotics have been used for their control and treatment. However, probiotics or prebiotics or their combination offer a potential alternative intervention strategy for improving the host health and preventing foodborne pathogen colonization/infections in reservoir. Further, bioengineered probiotics expressing bioactive products to achieve specific function is highly desirable. Recently, we over-expressed *mcra* (myosin cross-reactive antigen) gene in *Lactobacillu*s *casei* (Lc) and developed a bioengineered probiotics Lc + CLA which produce higher amounts of metabolites including conjugated linoleic acid (CLA). Furthermore, we also reported that prebiotic like components such as berry pomace (byproduct) phenolic extracts (BPEs) can enhance the growth of probiotics and improved the beneficial effects of probiotics. In this study, we evaluated the antimicrobial effect of modified Lc + CLA in combination of BPEs on growth, survival and pathogenesis of enterohemorrhagic *Escherichia coli* (EHEC).

**Results:**

In mixed culture condition, the growth of EHEC was significantly reduced in the presence Lc + CLA and/or BPEs. Cell-free cultural supernatant (CFCS) collected from Lc or Lc + CLA strain also inhibited the growth and survival of EHEC and the inhibitory effects of CFCSs against EHEC were enhanced in the presence of BPEs in concentration dependent manner. Interaction between EHEC and intestinal epithelial INT-407 cells were also altered significantly in the presence of either Lc or Lc + CLA strain or their CFCSs with or without BPEs. The expression of multiple virulence genes and physicochemical properties of EHEC were also altered when the bacterial cells were pretreated with CFCSs and/or BPEs.

**Conclusions:**

These results showed that diet containing bioactive Lc + CLA and natural prebiotic like component such as BPEs might be an effective way to prevent foodborne infections with EHEC.

## Background

Majority of enteric infections are foodborne and, in most case, bacteria are the major pathogenic agents followed by viruses, and then parasites. Among the enteric bacterial pathogens, Enterohemorrhagic *Escherichia coli* (EHEC) is one of the pre-dominants enteric bacterial pathogen in the USA and other developed countries. EHEC is frequently isolated from beef and other food products such as produce as well as recorded as a top ranked foodborne bacterial pathogenic agents in respect to mortality and morbidity [1, 2]. Enteric infection with EHEC also can lead to kidney failure due to the severe cytotoxic effect of EHEC known as Hemolytic Uremic Syndrome (HUS) [1]. This effect could be intensified due to the antibiotic therapy [2]. In addition, with high incidence of antibiotic resistance in EHEC [3], the development of novel bio-therapeutics against this specific enteric bacterial pathogen, is more crucial than ever.

Probiotic, as predominate part of gut microbial ecosystem, plays critical roles in maintaining the balance of human GI ecosystems and prevent the enteric infections by limiting colonization of enteric pathogen [4–6]. In addition, with host health promotion, probiotics showed several defensive or beneficial activities against pathogens including competitive exclusion, inhibition of bacterial protein synthesis, limiting quorum sensing, secretion of proteins, and their motility/mobility [4–8]. However, all these beneficial roles of probiotics against enteric bacterial pathogens generally depend on the ratio of probiotic within the gut ecosystems and the total amount of bioactive metabolites specifically short chain fatty acids and other acids produced by them.

On the other hand, the use of dietary plant phenolic extracts is becoming an attractive alternative therapy [9–11]. Fruit byproducts, especially blackberry (*Rubus fruticosus*) and blueberry (*Vaccinium corymbosum*) byproducts commonly known as pomace, contain bioactive phenolics including flavan, flavanone, flavones, glucuronides, glucosides, quinolones, catechol, coumarin, phenols, luteolines, tannins, quercetin, chlorogenic acid, ellagic acid, gallic acid, xanthoxic acid [12]. Recent reports have shown that berry pomace phenolic extracts (BPEs) are antimicrobial against a wide variety of enteric bacterial pathogens [13–16] and in the presence of BPEs, growth of beneficial bacterial/probiotic is enhanced with increased production of the bioactive metabolites [17–20].

Combination of probiotic and prebiotic, known as synbiotics, have emerged as a promising alternative treatment approach and can improve and maintain host health; beneficial effects depend largely upon the total quantity of probiotics and the amount and type of functional byproducts (proteins and peptides) they produce. In a recent study, we found that in the presence of the prebiotic-like component peanut flour, *Lactobacillus casei* (Lc) produced 100 times more linoleic acid (LA) than under normal condition and was able to outcompete several enteric bacterial pathogens [13, 21, 22]. On the basis of such observation, we have overexpressed the linoleate isomerase (myosin cross-reactive antigen, *mcra*) gene in a natural, sustainable Lc strain in order to enhance the production of conjugated linoleic acids (CLA) [23, 24] and verify the ability of this genetically engineered strain (Lc + CLA) to inhibit growth and infection of host cells by EHEC in vitro.

In this study, we assess the effect of genetically modified *L. casei* with increased production ability of CLA, known as Lc + CLA in combination with various concentration of BPEs against the growth and survival ability EHEC and its interaction with cultured human intestinal epithelial (INT-407) cells. Further, we also compare expression levels of EHEC virulence mediatory genes, and physicochemical properties in the presence or absence of the probiotic strains and/or bioactive phenolic extracts.

## Results

### Growth inhibition of EHEC in presence or absence of probiotic strains and/or pomace phenolic extracts

**Fig. 1:**
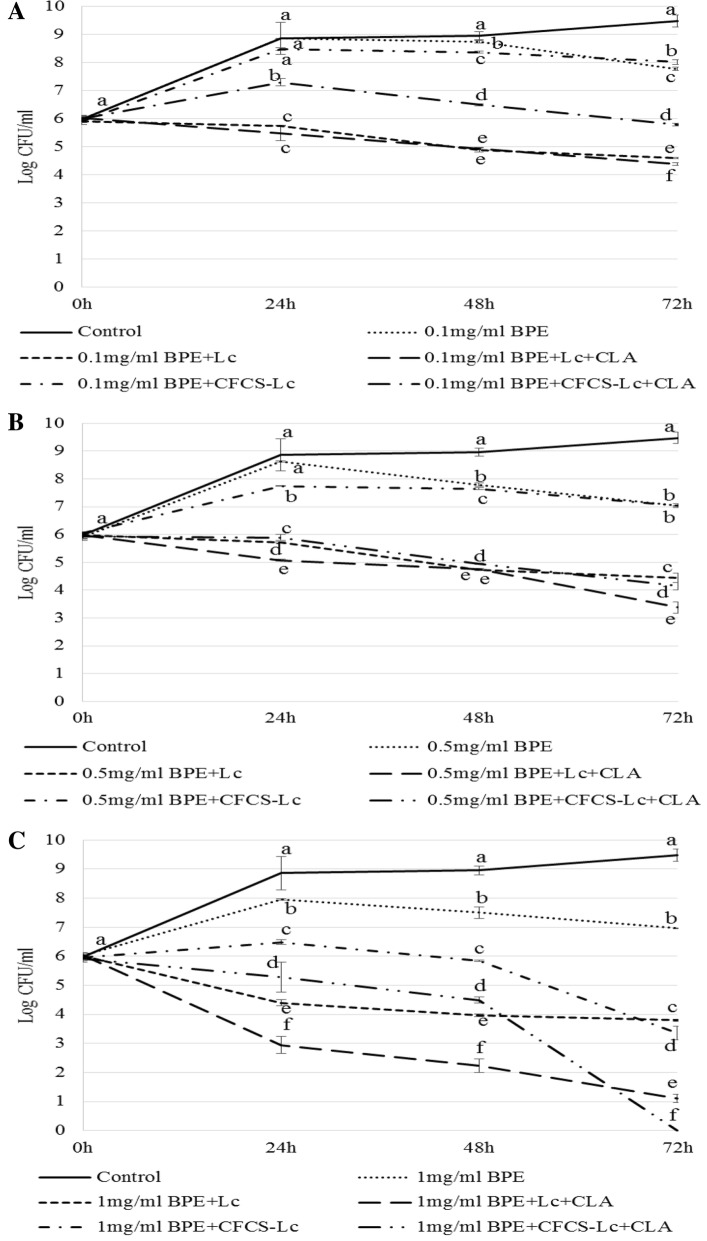
Growth pattern of EHEC (**A**–**C**) at various time (24, 48, and 72 h) points with or without treatments. Error bars indicate standard deviation from 6 parallel trails. Different letters (a–f) at each time point indicate the significant growth reduction when compared with single culture as a control and among the treatments at *p *< 0.05

To determine the effect of prebiotic like component BPEs, probiotic strain Lc or Lc + CLA or the metabolites produced by Lc or Lc + CLA in CFCSs on the growth inhibition of EHEC, we co-cultured EHEC with Lc or Lc + CLA or their CFCSs with different concentrations of BPEs [0.1 mg/ml gallic acid equivalent (GAE) or 0.5 mg/ml GAE or 1.0 mg/ml GAE]. In the presence of BPEs either of 0.1 mg/ml GAE or 0.5 mg/ml GAE, the growth of EHEC was not significantly reduced compared to the control but when the concentration of BPE was raised to 1.0 mg/ml GAE, the growth of EHEC was reduced significantly within 24 h (Fig. 1A–C). Further, either probiotic strain, Lc or Lc + CLA in combination with 0.1 mg/ml GAE or 0.5 mg/ml GAE or 1.0 mg/ml GAE of BPEs intensified the reduction of the growth of EHEC in a time dependent manner but the inhibitory effect of Lc + CLA in presence of 1.0 mg/ml GAE was observed at maximum level [> 8.0 logs colony forming unit (CFU)/ml reduction] than the other treatments (Fig. 1C). CFCS collected from of Lc (CFCS-Lc) or Lc + CLA (CFCS-Lc + CLA) in the presence of BPEs of different concentrations also showed inhibitory effects on the growth of EHEC (Fig. 1A–C). After 24 h, the reduction in the growth of EHEC was observed ranging from < 1.0 log CFU/ml (CFCS-Lc in presence of 0.1 mg/ml GAE of BPE) to > 3.0 logs CFU/ml (CFCS-Lc + CLA in presence of 1.0 mg/ml GAE of BPE). After 48 h of incubation, the growth of EHEC was reduced more than 3.0 logs CFU/ml by CFCS-Lc in presence of 1.0 mg/ml GAE of BPEs and more than 4 logs CFU/ml by CFCS-Lc + CLA in presence of BPEs of 1.0 mg/ml GAE concentration compared to control group (no CFCS or BPEs added to the growth medium). After 72 h of incubation, the most effective growth reduction of EHEC was observed by CFCS-Lc + CLA in presence of 1.0 mg/ml GAE of BPEs with no detectable growth (Fig. 1C).

### Alteration of EHEC adhesion ability in cultured mammalian cells

**Fig. 2:**
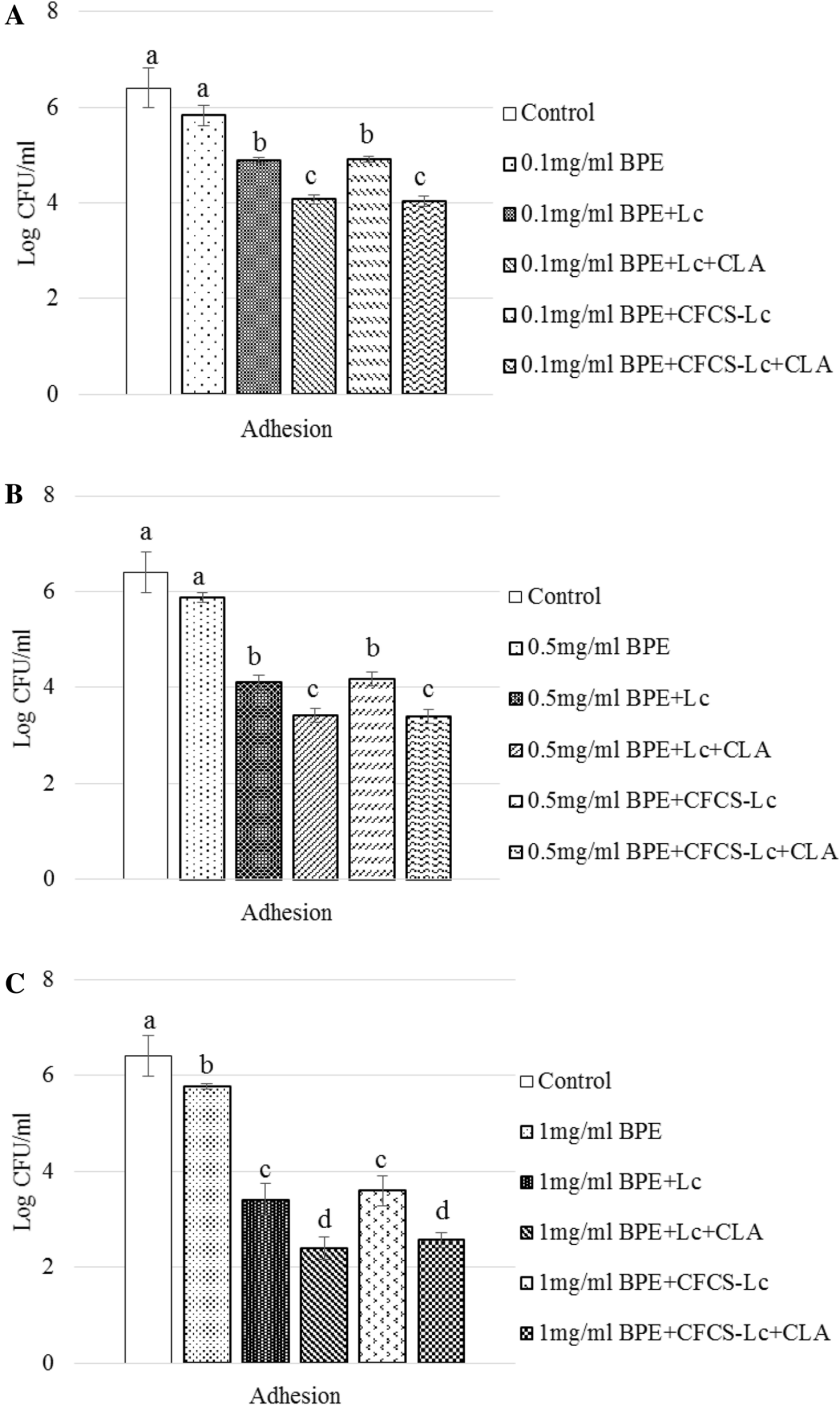
Adhesion of EHEC to INT-407 cells with or without pre-treatments (**A**–**C**) with probiotics (Lc or Lc + CLA) and their metabolites in CFCSs. Error bars indicate standard deviation from 6 parallel trails. Bars with different letters (a through d) are significantly different when compared with control and among the treatments at *p *< 0.05

The adhesion ability of EHEC to INT-407 cells in the presence of different concentrations (0.1 mg/ml GAE or 0.5 mg/ml GAE or 1.0 mg/ml GAE) of BPEs, in combination with Lc or Lc + CLA itself or their metabolites containing CFCSs (CFCS-Lc and CFCS-Lc + CLA) was reduced significantly from 0.5 log CFU/ml to 4.0 logs CFU/ml (Fig. 2A–C). BPEs (0.1 mg/ml GAE or 0.5 mg/ml GAE) without the probiotics or their CFCSs, numerically reduced the adhesion ability, though 1.0 mg/ml GAE reduced adhesion ability around 0.6 log CFU/ml, significantly (Fig. 2A–C) when compared to the control (only medium without any treatments).

### Alteration of EHEC physicochemical properties in presence of BPEs and/or CFCSs collected from Lc or Lc + CLA

We observed that pre-treatments of EHEC with all the treatments accept 0.1 mg/ml GAE of BPE, significantly increased the percentage of injured bacterial cells ranging from 36.33 to 58.97% when compared to control (without any treatments in growth media) (Table 1). The auto-aggregation capacity of EHEC decreased significantly by CFCS-Lc in presence of 1.0 mg/ml GAE of BPE or CFCS-Lc + CLA in presence of 0.5 mg/ml GAE or 1.0 mg/ml GAE of BPEs, and CFCS-Lc + CLA in presence of 1.0 mg/ml GAE of BPE was the most effective when compared to control group. The auto-aggregation capacity of EHEC was reduced numerically by 0.1 or 0.5 or 1.0 mg/ml GAE of BPE, CFCS-Lc in presence of 0.1 mg/ml GAE or 0.5 mg/ml GAE of BPE, CFCS-Lc + CLA in presence of 0.1 mg/ml of GAE but not significantly (Table 1). We also found that cell surface hydrophobicity of EHEC was reduced significantly with all the pre-treatments ranging from 4.65 to 1.45% accept 0.1 mg/ml GAE of BPE. Also, BPE of 0.1 mg/ml GAE concentration reduced the cell surface hydrophobicity of EHEC to 13.1% when compared to control group but only numerically (Table 1).

**Table 1:** Physicochemical properties of EHEC treated with BPEs/CFCSs collected from overnight culture of Lc/Lc + CLA

Treatment	Auto-aggregation (%)	Hydrophobicity (%)	Injured cell (%)
Control	5.89 ± 1.47*^,a^	13.1 ± 2.16^a^	16.52 ± 5^a^
0.1 mg/ml GAE	5.38 ± 1.61^a^	9.87 ± 2.52^ab^	26.84 ± 5.07^ab^
0.1 mg/ml GAE + CFCS-Lc	4.65 ± 2.7^a^	8.28 ± 2.49^bc^	41.4 ± 5.57^bc^
0.1 mg/ml GAE + CFCS-Lc + CLA	2.91 ± 1.52^ab^	4.57 ± 2.06^bc^	50.35 ± 3.93^c^
0.5 mg/ml GAE	4.45 ± 1.34^a^	8.38 ± 1.98^b^	36.33 ± 6.06^b^
0.5 mg/ml GAE + CFCS-Lc	3.41 ± 1.56^a^	6.85 ± 1.77^bc^	45.9 ± 4.77^bc^
0.5 mg/ml GAE + CFCS-Lc + CLA	2.18 ± 1.21^b^	3.5 ± 1.2d^c^	53.2 ± 7.01^c^
1.0 mg/ml GAE	3.69 ± 0.86^a^	6.36 ± 1.74^bc^	45.34 ± 5.42^bc^
1.0 mg/ml GAE + CFCS-Lc	2.78 ± 1.41^b^	4.8 ± 1.54^c^	49.92 ± 6.47^bc^
1.0 mg/ml GAE + CFCS-Lc + CLA	1.45 ± 0.02^b^	2.89 ± 1.14^c^	58.97 ± 3.45^c^

*Values indicate mean ± standard deviation and means with different letters (a–c) within the same column are different when compared with control and among the treatments at p < 0.05

### Disruption of biofilm formation of EHEC with BPEs and/or CFCSs collected from Lc or Lc + CLA

**Fig. 3:**
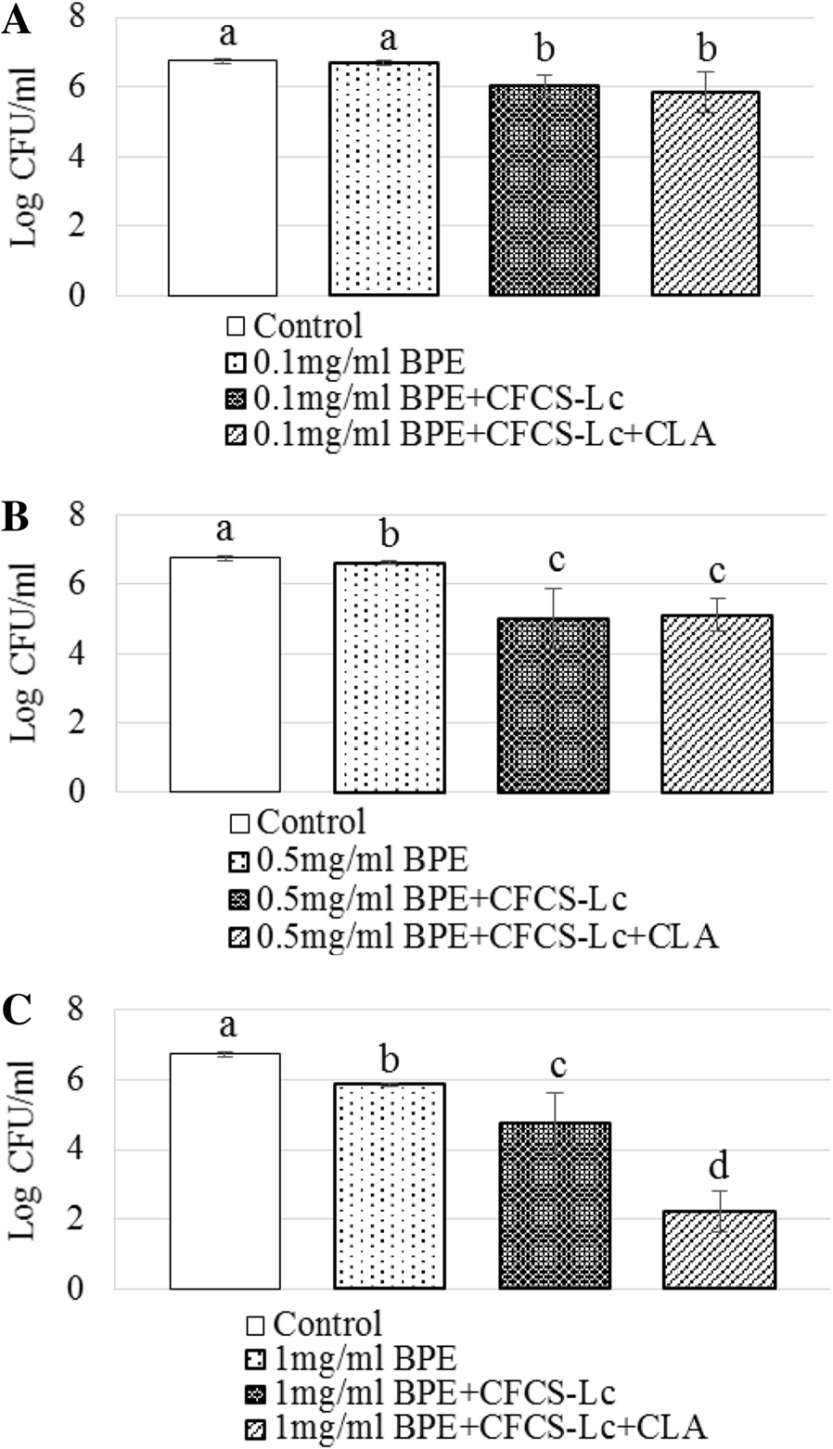
Biofilm formation pattern of EHEC (**A**–**C**) treated with BPEs alone or CFCSs collected from Lc or Lc + CLA grown in the presence of BPEs. Error bars indicate standard deviation from 6 parallel trails. Different letters (a–d) indicate the significant reduction in biofilm formation when compared with control and among the treatments at *p *< 0.05

Biofilm formation is the process of attachment to a surface which can affect the growth rate and gene transcription ability of a bacteria and when EHEC was incubated in presence of BPEs (0.1 mg/ml GAE or 0.5 mg/ml GAE or 1.0 mg/ml GAE) without CFCSs (collected from overnight culture of Lc or Lc + CLA) the biofilm formation ability was reduced significantly around 1.0 log CFU/ml. BPEs in combination with CFCS-Lc or CFCS-Lc + CLA also reduced the biofilm formation ability of EHEC when compared to the control group (growth media without treatment), significantly from < 1.0 log CFU/ml to > 4.4 logs CFU/ml and CFCS-Lc + CLA was more effective in the reduction of the biofilm formation in presence of same concentration of BPE in comparison with CFCS-Lc (Fig. 3A).

### Effect of CFCSs collected from Lc or Lc + CLA with or without BPEs on EHEC virulence gene expression

**Fig. 4:**
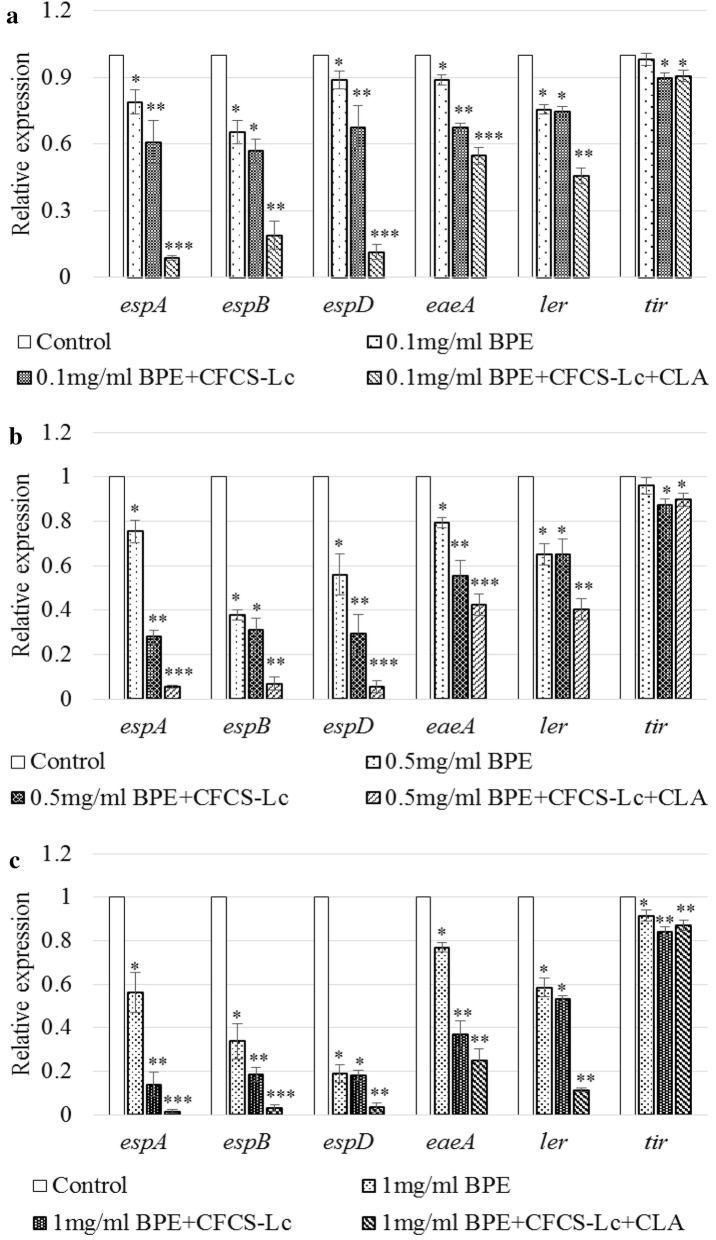
Relative expression of different virulence genes of EHEC (**a**–**c**) treated with BPEs alone or CFCSs collected from Lc or Lc + CLA grown in the presence of BPEs (0.1 mg/ml GAE or 0.5 mg/ml GAE or 1.0 mg/ml GAE). Error bars indicate standard deviation from 6 parallel trails. Bars with different number of asterisks (*) are significantly different at *p *< 0.05

Expression of *espA* gene of EHEC, which is responsible for encoding secreted protein related to signal transduction leading to A/E lesion formation, was significantly down-regulated at a range from 1.2- to 62.5-folds by either CFCS-Lc or CFCS-Lc + CLA collected from Lc or Lc + CLA in the presence of different concentrations of BPEs (Fig. 4a–c). The expression level of *espB* and *espD* genes which are also involved in signal transduction leading to A/E lesion formation were down regulated significantly ranging from 1.1- to 28-folds in the treatment assay. The expression level of *eaeA* encoding intimin protein required for intimate attachment was also down regulated significantly more than 1.1-folds to more than 3.9-folds in the assay. The expression level of locus of enterocyte effacement (LEE)-encoded regulator, *ler* was downregulated significantly > 1.3- to > 8.5-folds by all the concentrations of BPEs with or without CFCS-Lc or CFCS-Lc + CLA. The expression level of *tir* responsible for translocated intimin receptor was also downregulated around onefold by different concentrations of BPEs with or without CFCS-Lc or CFCS-Lc + CLA (Fig. 4a–c).

## Discussions

In this study, the combined effect of Lc + CLA, the probiotic strain with enhanced ability of conjugated linoleic acid (CLA) production, and bioactive phenolic compounds, BPEs extracted from blackberry and blueberry pomaces (byproducts), on the growth, survival and physicochemical properties of EHEC and in alteration of its interactions with human intestinal epithelial (INT-407) cells was investigated. BPE which showed its anti-oxidation and modulation ability of gut microbiome positively in our previous chicken trial [25], was tested in this study to amplify the beneficial effects of Lc or Lc + CLA.

We observed the synergistic effect of Lc or Lc + CLA and BPEs enhance the inhibiting ability of the growth of EHEC but the effect of BPE depends on concentration and duration of treatment period. These findings satisfy the previous reports in which we observed that CLA overproducing strain, Lc + CLA and its metabolites containing CFCSs could alter the pathogenesis of several enteric bacterial pathogens including EHEC, *Salmonella* and *Campylobacter jejuni* [23, 24]. It has been also reported that CLA improved host gut health and immunity by protecting against inflammation and also play important role in metabolic pathways and in balancing gut microbial ecosystem [26, 27]. Further, researchers have reported that secondary plant metabolites containing bioactive phenolic extracts possess antimicrobial and anti-oxidant properties [28, 29]. In our laboratory, we also found that phenolic extracts from blueberry and blackberry pomace could inhibit the growth and survival of various enteric bacterial pathogens including *Salmonella* and *Campylobacter* and altered the virulence properties of these pathogens and their interaction with host cells [12, 15, 30]. In our in vivo study, we also found that BPE can reduce the colonization of enteric bacterial pathogen *Campylobacter* in chicken gut on concentration dependent manners and a very trace amount (0.1 GAE mg/ml) of BPEs also act as a growth promoter in chicken [31]. In this study, we found the combined effects CLA over-producing *Lactobacillus* strain with various concentration of BPEs can inhibit the growth and alter different pathogenic traits of EHEC at higher efficacy compared to BPEs at the same concentration alone.

In this current study, we also observed that CFCSs collected from either Lc or Lc + CLA could modify the growth and pathogenesis of EHEC and effect of CFCSs on EHEC growth reduction may not only depend on medium acidification, as growth of EHEC at a wide range of pH was previously observed [32]. As anti-pathogenic traits of phenolic compounds and Lc + CLA along with other metabolites in CFCSs showed an intensive effect on EHEC, it can be inferred that both metabolites produced by Lc or Lc + CLA and bioactive phenolic components of berry pomace could act synergistically, also found previously [33].

As attachment is considered to be important virulence properties, in this study we observed that adhesion efficacy of EHEC was reduced significantly in cultured mammalian intestinal epithelial cells, INT-407 in co-culture with Lc or Lc + CLA or treated by the CFCSs obtained from overnight culture of Lc or Lc + CLA in presence of BPEs. Higher concentrations of BPEs alone could reduce the adhesion efficacy significantly compared to growth media without any supplement. It has been reported before that similar carbohydrate-binding specific proteins are displayed on *Lactobacillus* spp. surface and may involve in decreasing the adhesiveness of enteric bacterial pathogens by pre-occupying the surface receptors on host cells [34]. Researchers have also reported that CFCSs, collected from various condition of *Lactobacillus* spp. also restricted the cells adhesion to host epithelial cells by different enteric bacterial pathogens [4, 35]. To further investigate the attenuated bacterial virulence at gene expression level, we observed that the expression level of different virulence genes. The *ler* is the main transcriptional regulator for EHEC that modulates all effectors especially *espA*, *espB*, *espD*, *eaeA* and *tir* for attaching and effacing of the bacteria [36, 37] and were down regulated in the presence of the treatments of this study. This finding indicated that the down-regulation of the transcriptional regulator repressed the expression of effector genes for bacterial attachment, and therefore directly related to the reduction of EHEC-host cell interactions and other pathogenicity related traits.

Several groups of researchers reported positive relation among hydrophobicity, auto-aggregation and cell association activities [38–40]. In this study, combined treatments with CFCSs from Lc/Lc + CLA in presence of BPEs resulted in decreased hydrophobicity and auto-aggregation, which might impact on the reduction of adhesiveness in EHEC into INT-407 cells [12, 22, 41]. We also observed deferred ability of biofilm formation by EHEC with the combined treatments of CFCSs collected from either Lc or Lc + CLA in presence of different concentrations of BPEs. A positive association between bacterial auto-aggregation, cell surface hydrophobicity to biofilm formation ability has also been reported previously and our findings agreed with other study [42]. Injured but viable EHEC cell percentage was increased significantly by all the treatments and the effect intensified with the higher concentrations of BPEs or higher concentrations of bioactive metabolites, as higher concentration of BPEs were more efficient or CFCS from Lc + CLA was more effective compared to CFCS from Lc in presence of the same concentration of BPE. We hypothesized that the ratio of injured bacterial cells depends on the anti-pathogenic metabolites present in the treatments.

## Conclusion

Probiotics specifically linoleic acid over-producing *Lactobacillus* strain, Lc + CLA, in the presence of BPEs exhibited rigorous effects on EHEC pathogenesis. Further, the growth medium supplemented with trace amount of BPEs stimulated the growth of probiotic strains [33], and also intensified the inhibitory effect of CFCSs collected from Lc + CLA on growth and survival of EHEC and altered the INT-407 cells-EHEC interactions. The promising roles of the combination of the genetically engineered probiotic strain, Lc + CLA and bioactive BPEs in controlling foodborne infection with EHEC, particularly growth inhibition, physicochemical properties alteration, disruption of EHEC-host cell interactions, and its virulence genes suppression indicated the possibility to make this synbiotic potential alternative to preventive and/or therapeutics for EHEC infection in human. In future, the effectiveness of the synbiotic is needed to confirm in appropriate animal model.

## Materials and method

### Bacterial strains and growth conditions

In this study, enterohemorrhagic *Escherichia coli* EDL933 (EHEC) (ATCC 700927) was grown on Luria-Bertani (LB) agar (EMD Chemicals Inc., USA) for 18 h at 37 °C under aerobic conditions (Thermo Fisher Scientific Inc., USA). Probiotic strains, *Lactobacillus casei* (Lc) (ATCC 334) and lineolate over-expressed bioactive *L. casei* (Lc + CLA) [23, 24] were grown on de Man Rogosa Sharpe (MRS) agar (EMD Chemicals Inc., USA) overnight at 37 °C under aerobic condition with 5% CO_2_ (Thermo Fisher Scientific Inc., USA).

### Preparation of pomace phenolic extracts

Commercial blackberry and blueberry pomaces was donated by Milne Fruit Products Inc., Prosser, WA, USA, and BPE was extracted following the protocol previously reported [13]. Spectrophotometric method was used to measure the concentration of BPE and expressed as GAE [43]. BPE was comprised of blackberry and blueberry pomace extracts at 1:1 v/v ratio for this study.

### Mammalian cell and culture conditions

Human intestinal epithelial (INT-407) (ATCC CCL-6) cells were cultured at 37 °C, standard condition (5% CO_2_) in Dulbecco’s Modified Eagle Medium (DMEM) (Corning Cellgro, USA) supplemented with 10% heat-inactivated Fetal Bovine Serum (FBS) (Corning Cellgro, USA) and 50 µg/ml of gentamycin (Lonza, USA). For monolayer preparation, INT-407 cells were seeded in 24-well culture plate (Greiner Bio-one Inc., USA) at 2 × 10^5^ cells/ml and maintained following the standard protocol described as above to form > 90% confluence monolayer. Before use, the monolayers were washed with phosphate buffer saline (PBS) three times and immersed in antibiotic free DMEM supplemented with 5% heat-inactivated FBS [24].

### Cell free culture supernatant

Cell free culture supernatant (CFCS) of overnight liquid cultures of Lc, and Lc + CLA were collected following the method previously reported by our laboratory [21] and collected CFCSs were filtered and stored at 4 °C.

### Growth inhibition assay

Inhibition of EHEC growth was carried out in presence of different concentrations of BPEs (0.1 mg/ml GAE or 0.5 mg/ml GAE or 1.0 mg/ml GAE) and/or Lc or LC + CLA. All bacterial strains were grown on selective respective agar plates following the method described above. A volume of 400 µl of Lc or Lc + CLA bacterial suspension containing approximately 10^7^ CFU/ml was mixed with equal volume of EHEC suspension containing approximately 10^6^ CFU/ml in 3.2 ml of LB broth and incubated at 37 °C in presence of different concentrations (0.1 mg/ml GAE or 0.5 mg/ml GAE or 1.0 mg/ml GAE) of BPE. Serial dilutions were performed in PBS, followed by plating on LB agar for EHEC at 0, 24, 48, and 72 h time points. For inhibition assay with CFCSs, instead of live Lc or Lc + CLA, 400 µl of CFCS-LC (collected from Lc) or CFCS-Lc + CLA (collected from Lc + CLA) with/without BPEs were mixed with equal volume of EHEC suspension containing approximately 10^6^ CFU/ml in 3.2 ml of LB broth and incubated at 37 °C and counted following the above mentioned plating methods at 0, 24, 48 h and 72 h time points.

### Cell adhesion assay

Adhesion of EHEC to INT-407 cell was performed following the method described previously [21, 24]. Briefly, INT-407 cell monolayers were pre-treated with 100 µl DMEM (control), different concentrations of BPEs (0.1 mg/ml GAE or 0.5 mg/ml GAE or 1.0 mg/ml GAE) or with probiotics or their CFCS in presence of different concentrations of BPEs (0.1 mg/ml GAE or 0.5 mg/ml GAE or 1.0 mg/ml GAE) for 1 h (triplicates). After pre-treatment, 100 µl of EHEC, bacterial suspension with multiplicity of infection (MOI) of 10 (2 × 10^6^ CFU/ml) were inoculated into each well, followed by 2 h incubation, lysis by 0.1% Triton X-100 for 15 min, serial dilutions and plating for quantification.

### Physiological properties of EHEC in the presence of probiotics or their CFCS and/or BPEs

Changes of physicochemical properties including cell surface hydrophobicity, auto-aggregation and injured cell ratio of EHEC were evaluated following the methodologies previously described [16] with modifications in culture condition. In brief, EHEC was grown in LB broth or LB broth with BPEs (0.1 mg/ml GAE or 0.5 mg/ml GAE or 1.0 mg/ml GAE) or CFCSs collected from Lc or Lc + CLA in combination with BPEs at 37 °C for 18 h.

### EHEC biofilm formation

The ability of EHEC to form biofilms on glass surfaces in the absence or presence of CFCSs and/or BPEs was performed following the method previously described [16]. Briefly, 100 µl of EHEC, containing approximately 5 × 10^5^ CFU/ml, was inoculated in triplicate in wells of 6-well plates (Corning, USA) containing 22 × 22 mm^2^ glass slides. Wells containing LB broth (control) or LB broth supplemented with different concentrations of BPEs (0.1 mg/ml GAE or 0.5 mg/ml GAE or 1.0 mg/ml GAE), or CFCSs with BPEs were incubated for 48 without shaking at 37 °C. Then, the glass slides were rinsed with PBS for five times and bacterial cells were recovered using sterile cell scraper (VWR, USA) from the glass surface and enumerated.

### RNA extraction, cDNA synthesis and quantitative RT-PCR assay

EHEC was grown in the absence or presence of BPEs (0.1 mg/ml GAE or 0.5 mg/ml GAE or 1.0 mg/ml GAE) or/and CFCSs collected from Lc or Lc + CLA and RNA was extracted according to the protocol of ZR Bacterial RNA MiniPrep kit (Zymo Research Corp., USA). The RNA quantification was carried out using NanoDrop spectrophotometer (Thermo Scientific Inc., USA) and cDNA synthesis was performed according to the protocol provided by Quanta Biosciences, USA. The custom-synthesized oligonucleotide primers for *espA*, *espB*, *espD*, *eaeA*, *ler*, *tir* of EHEC were purchased from Eurofins MWG Operon, USA and methodology previously described [44] was followed to perform the qRT-PCR assay.

### Statistical analysis

Collected data were analyzed by the Statistical Analysis System software (SAS Institute Inc., USA). The one-way analysis of variance (ANOVA) for each single time point followed by Tukey’s test was used to evaluate the various treatments and significant differences among control and treatments were determined based on significant level of 0.05.

## Data Availability

The raw data and/or analysis of the current study are available from the corresponding author on reasonable request.

## Abbreviations

BPE: berry pomace (byproduct) phenolic extract
CFCS: cell-free cultural supernatant
CFU: colony forming unit
CLA: conjugated linoleic acid
DMEM: Dulbecco’s Modified Eagle Medium
EHEC: enterohemorrhagic *Escherichia coli*
FBS: fetal bovine serum
GAE: gallic acid equivalent
HUS: hemolytic uremic syndrome
LA: linoleic acid
LB: Luria–Bertani
Lc: *Lactobacillu*s *casei*
Lc + CLA: genetically engineered strain of *Lactobacillu*s *casei*
LEE: locus of enterocyte effacement
*Mcra*: myosin cross-reactive antigen
MRS: de Man Rogosa Sharpe
PBS: phosphate buffer saline
